# Bistable persistent spiking of layer II and layer V medial entorhinal cortical neurons during theta frequency oscillations in vitro

**DOI:** 10.1186/1471-2202-13-S1-P124

**Published:** 2012-07-16

**Authors:** Nathan W Schultheiss, Michael E Hasselmo

**Affiliations:** 1 Psychology; Center for Memory and Brain, Boston University, Boston, MA 02215, USA

## 

In the presence of muscarinic cholinergic modulation *in vitro*, persistent spiking neurons of medial entorhinal cortex (mEC) exhibit bistability in that they can be switched from quiescence to spiking by a brief depolarizing input. The spiking state is thought to be maintained by a positive feedback loop between a nonspecific, calcium-sensitive cationic current (ICAN) carried by canonical transient receptor potential (TRPC) channels and heightened intracellular calcium levels sustained during sufficiently fast spiking. Bistable persistent spiking (PS_B_) has been observed across layers and cell types in mEC and has been implicated in working memory and grid cell function, so cholinergic modulation of TRPC channels represents a potentially fundamental mechanism shaping the spiking output of mEC neurons. However, spiking of mEC neurons during navigation and goal directed behavior *in vivo* is strongly theta modulated (4-12 Hz), and it is not known how the PS_B_ mechanism may interact with theta oscillations. In the present study we evaluated the effect of muscarinic modulation on input-output processing of mEC neurons to tonic, slowly varying, and oscillatory inputs. First, we investigated the frequency to injected current (FI) relationship using step and ramp current injection protocols in the presence of the muscarinic agonist carbachol (CCh). FI measures made following suprathreshold pre-pulse current injections exhibited higher spike frequencies than when measured following subthreshold pre-pulses, illustrating a spike-history dependence of the FI relationship. The instantaneous spike frequency measured during a range of current steps showed a positive slope for most PS_B_ neurons, indicating that ICAN acts as a reverse spike frequency adaptation mechanism. In response to linearly increasing ramp current stimuli, mEC neurons showed a steep acceleration followed by a plateau of spike frequency. Spike frequency hysteresis during decreasing ramp stimuli demonstrated significant adaptation for higher input levels, but spiking was often maintained at lower levels of injected current. Next, using different combinations of theta frequency sinewave current injections and DC holding currents, we were able to generate a range of spiking patterns where spikes were elicited on different proportions of theta cycles and at different phases of the theta input. With muscarinic activation, spiking was elicited on a higher proportion of theta cycles and at earlier phases of the oscillatory input for lower levels of tonic current input. A subset of recorded neurons exhibitted repeated sequences of intrinsic phase precessing spiking behavior. Similar to the plateau in spike rate observed with ramp stimuli, in response to stimuli composed of sinewaves superimposed on ramps, spiking phase rapidly advanced and maintained an 'early-phase plateau' as DC current continued to increase. Taken together, we demonstrate that muscarinic modulation of TRPC channels determines the gain of the frequency to injected current relationship of mEC neurons, interacts with adaptation mechanisms to generate spike history dependencies of spike rate including hysteresis, and shapes the distribution of spiking phases relative to oscillatory input. We propose that increasing activation of ICAN by spike-triggered calcium influx as an animal enters a firing field may provide a cellular mechanism contributing to theta phase precession of mEC grid cells.

